# Avian migration clocks in a changing world

**DOI:** 10.1007/s00359-023-01688-w

**Published:** 2024-02-02

**Authors:** Barbara Helm, Miriam Liedvogel

**Affiliations:** 1 Swiss Ornithological Institute, Bird Migration Unit, Seerose 1, CH-6204 Sempach, Schweiz; 2 Institute of Avian Research, An Der Vogelwarte 21, 26386 Wilhelmshaven, Germany

**Keywords:** Circannual, Circadian, Navigation, Migration programme, Climate change, Migratory restlessness

## Abstract

Avian long-distance migration requires refined programming to orchestrate the birds’ movements on annual temporal and continental spatial scales. Programming is particularly important as long-distance movements typically anticipate future environmental conditions. Hence, migration has long been of particular interest in chronobiology. Captivity studies using a proxy, the shift to nocturnality during migration seasons (i.e., migratory restlessness), have revealed circannual and circadian regulation, as well as an innate sense of direction. Thanks to rapid development of tracking technology, detailed information from free-flying birds, including annual-cycle data and actograms, now allows relating this mechanistic background to behaviour in the wild. Likewise, genomic approaches begin to unravel the many physiological pathways that contribute to migration. Despite these advances, it is still unclear how migration programmes are integrated with specific environmental conditions experienced during the journey. Such knowledge is imminently important as temporal environments undergo rapid anthropogenic modification. Migratory birds as a group are not dealing well with the changes, yet some species show remarkable adjustments at behavioural and genetic levels. Integrated research programmes and interdisciplinary collaborations are needed to understand the range of responses of migratory birds to environmental change, and more broadly, the functioning of timing programmes under natural conditions.

**Supplementary Information:**

The online version contains supplementary material available at 10.1007/s00359-023-01688-w.

## Introduction: migration across timescales

**Fig. 1:**
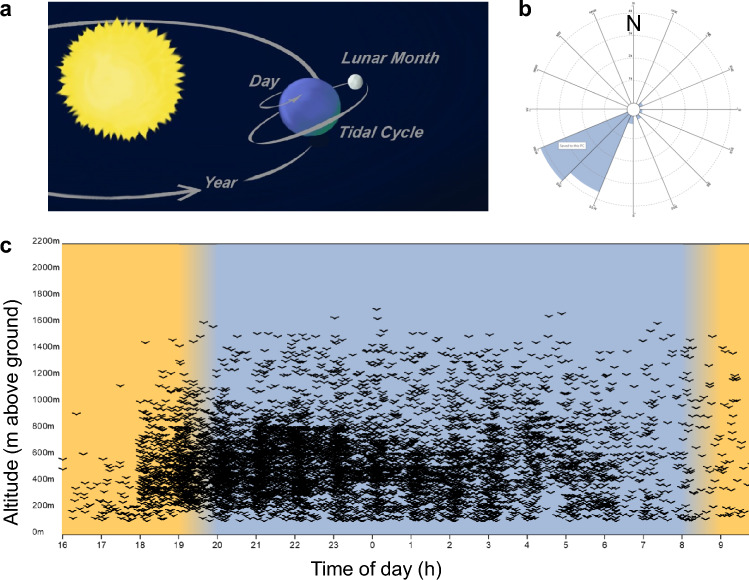
Timing of migration. (**a**) Migration, as a periodic process, is linked to highly predictable planetary movements on timescales of a year, a day, and of variants of lunar and tidal rhythms; image: Edda Starck. Echos from radar investigations show that avian migration is highly synchronized in space (**b**) and time (**c**). Bird flights (echos drawn as pictograms in (**c**)) cluster at night (blue background) and are strongly directional (blue bar showing nocturnal direction in (**b**)). Echos collected by dedicated vertical-looking radar in Sempach, Switzerland, on 17 October 2023; for methods, see (Shi et al. 2021)

Migration, the periodic arrival of animals at, and disappearance from, a given location, has long fascinated human observers. The patterns of temporal regularity were in some cases so striking that they were used to time various cultural practices, such as sowing seeds upon the migrants’ seasonal returns. Today’s observation tools and years of research efforts demonstrate an even broader natural phenomenon of migration than traditionally assumed. While definitions of migration vary (Dingle 1996), we use the term to describe coordinated, directed, periodic back-and-forth movements between at least two locations, whereby this alternation can occur within an individual or within a population (i.e., across generations). Thus defined, migration takes place in many taxa and on timescales linked to planetary cycles (Fig. 1; for an exception, see (Reynolds et al. 2014)).

Migrations are rhythmic on several timescales. On the scale of a day, diel vertical migration (DVM) of marine zooplankton occurs in massive volume over short distances, and may constitute earth’s greatest migration in terms of biomass (Berge et al. 2009). DVM is thought to coordinate foraging during light hours in surface water where phytoplankton photosynthesizes, and retreating during the night to lower depths where predation risk is reduced. In the marine realm, migrations also frequently occur on moon-linked time-scales, i.e., over lunar and tidal cycles (Tessmar-Raible et al. 2011). By automated acoustic monitoring, Last and colleagues (Last et al. 2016) showed that throughout the Arctic, zooplankton displays rhythmic movements during the polar night with periodicities of both the lunar day (i.e., 24.8 h) and the lunar month (29.5 d).

Perhaps the most evident migrations are annual return movements, ultimately due to earth orbiting around sun on a tilted axis (Foster and Kreitzman 2009) (Fig. 1). Animals migrate annually between areas that are temporarily conducive to reproduction, and areas that sustain them when breeding areas become inhospitable. Such annual shifts in habitat suitability could be due, for example, to scarcity of food or water, or to seasonally harsh climates. Amongst the best-known movements may be those of birds that migrate in flocks during the day. Yet migrations of other taxa are similarly spectacular, such as ungulate mammals moving through Serengeti, Monarch butterflies crossing between Mexico and Canada, or salmonid fish moving from the oceans to inland rivers (Horn and Narum 2023; Froy et al. 2003). Improved observation methods allow us to discover many further facettes and ecological impacts of movements, e.g., mass migrations of hoverflies (Syrphidae) that provide key ecosystem services across their range (Wotton et al. 2019).

Accurately orchestrated rhythmicity on multiple scales can be important for successful migrations. Birds perform broadly synchronized movements during pre-breeding (“spring”) and post-breeding (“autumn”) migrations. In addition, during migration season most species also alter their diel activity patterns, carrying out nocturnal flights while being predominantly diurnal for the remaining year. Thus, avian migration is typically synchronized tightly on both annual and diel timescales (Fig. 1).

Many migrations involve predictions over a broad spectrum of time and space. Rather than just dispersing or evading unsuitable environmental conditions, animals direct their movement towards locations which can be expected to be more suitable in the near future. Movements are thus often anticipatory, e.g., when animals leave a still prolific location before conditions deteriorate. Successful migration must master two challenges. The first is keeping track of time, sometimes over vast distances where cues to environmental conditions in remote target areas are absent or misleading (Åkesson and Helm 2020). The second challenge is to accurately navigate between distinct, often remote locations. Navigation can entail seasonal adjustment of direction depending on the phase of migration, and time-dependent interpretation of reference cues used for compass orientation such as the position of sun (i.e., solar azimuth; Kramer 1949; Froy et al. 2003)).

It is thus little wonder that research on biological rhythms has paid detailed attention to understanding how animal migration is coordinated and fine-tuned across different scales (e.g., Aschoff 1955; Froy et al. 2003; Gwinner 1986; Last et al. 2016; Rowan 1926)). Biological clocks play indeed central roles for migratory timing and navigation. For example, for DVM, researchers recently reported endogenous circadian migration and corresponding rhythms in metabolic activity and clock gene expression (Häfker et al. 2017). At the other extreme of the spatio-temporal spectrum, avian intercontinental migration is at least in some species based on endogenous circannual rhythms (i.e., rhythms with period lengths of ca. one year; Gwinner 1996b, 1986)). Given the timescale and distances covered, demands on clock mechanisms are particularly high for annual long-distance migration, on which we here focus. We furthermore base our review mostly on findings from long-distance migratory songbirds and in some cases from waders (charadriiforms). Partly due to the relative ease of study in captivity and in the wild, most available information on timing mechanisms comes from these groups. Given the few data, and since an in-depth discussion of parallels and differences of migration timing of other taxa is beyond the scope of this article, we acknowledge that extrapolation of our assessment to other taxa is speculative.

## Avian migration

### Phenomenon

Annual migrations of birds have intrigued chronobiologists from early days. Several aspects can hardly be explained without invoking clocks. For example, during their non-breeding season, migrants may experience local summer conditions on non-breeding grounds while local resident species breed. Still, migrants do not activate their own reproductive system in these areas. Rather, they leave non-breeding areas in time to reach their remote natal areas, where they then breed at the locally appropriate time (Hamner and Stocking 1970). This seems puzzling as photoperiod (i.e., the daylight fraction of a day), which otherwise provides reliable annual information, can offer little explanation of migratory timing. Photoperiod is drastically altered by migration across latitudes, and varies widely in species that continue to move in their non-breeding areas (Åkesson and Helm 2020; Gwinner 1996b). William Rowan, who pioneered photoperiodism, thus concluded that additional mechanisms must be involved for remote timing that also buffers against untimely daylength cues (Rowan 1926). Similarly, Jürgen Aschoff, a pioneer in biological rhythms research, speculated that migratory birds should possess long-term internal clocks that entrain to relevant timing cues (i.e., Zeitgebers; (Aschoff 1955)).

Use of long-term rhythms carries the challenge of requiring precision while remaining responsive to environmental conditions during the often risky journey (Åkesson and Helm 2020). For example, migration must be punctual but take place with sufficient energy reserves, and ideally under clement conditions with supportive winds (Newton 2008). Thus, avian migration requires clocks that simultaneously provide rigorous timing and heightened responsiveness to various environmental factors. Investigating how this balance is kept is difficult for a behaviour that takes place in midair and often spans continents. It is still thus largely mysterious how birds know when to depart, which direction to fly, when to stop, and when to return.

#### Migratory restlessness reveals an inherited migration programme for space and time

**Fig. 2:**
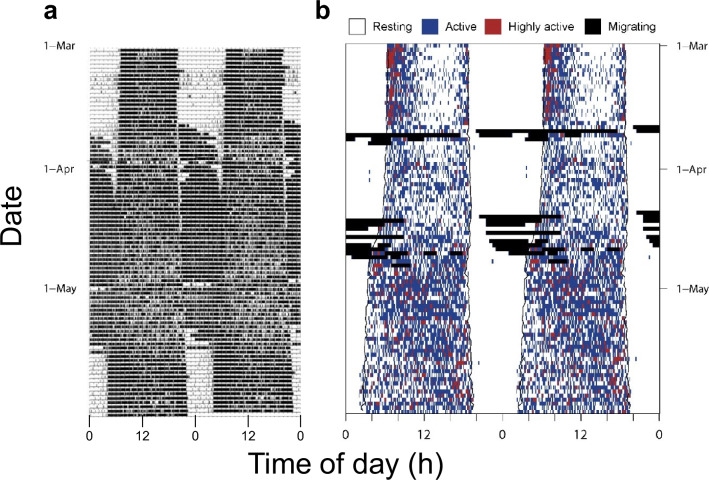
Day–night activity patterns of a caged migratory stonechat *Saxicola maurus* (**a**) and free-flying Tawny pipit *Anthus campestris* (**b**) during spring. Data in (**a**) were analyzed by Van Doren et al. (2017), those in (**b**) by Briedis et al. (2020). Double-plotted actograms show activity (dark) and rest (light) prior to, during, and after spring migration. In both actograms, every line represents a new day of recorded behaviour, shown after the repeated plotting of the recording of the preceding 24 h day. Activity from passive motion sensors in (**a**) is shown in black. Recordings in (**b**) are based on accelerometry, allowing for distinct colour-coding of activity, high activity and migration

The habit of most migratory birds to carry out their journeys at night (Fig. 1) has been instrumental for research on annual and diel timing. Bird keepers have long observed that captive migrants extend their activities into the night once the migratory season approaches, by flying, hopping and whirring their wings (e.g. (Berthold 2001)) (Fig. 2a). This so-called migratory restlessness (or German “Zugunruhe”) behaviour is generally coterminous with migration of free-living conspecifics. With the introduction of video-recording and automatic monitoring cages equipped with perch-switches and motion sensors, Zugunruhe became a widely, but not unanimously (e.g., Farner 1955; Helms 1963)), accepted proxy to study migration of wild birds. Its study provided the opportunity for experimental approaches that manipulate environmental conditions in a controlled manner. Of particular importance for biological rhythms research were studies under constant conditions, i.e., when birds were kept isolated from environmental cues by unchanging photoperiod, temperature, housing and food availability (Gwinner 1986; Berthold 2001; Newton 2008). Birds expressed Zugunruhe under simulated natural light conditions, under constant photoperiodic cycles (e.g., of 12 h light and 12 h darkness per day, LD 12:12 h), and under continuous dim light (e.g., Holberton and Able 1992; Gwinner 1986)).

Migratory restlessness that persisted under constant conditions revealed endogenous timing mechanisms operating simultaneously on annual and diel timescales. A key figure, Eberhard Gwinner, studied small songbirds (*Phylloscopus* warblers) in captivity under natural and constant daylengths in Germany and in their African winter quarters (Gwinner 1967, 1969). His studies mostly examined hand-raised, naive (i.e., inexperienced) birds that had never migrated in the wild and had no prior knowledge of non-breeding grounds. Patterns of Zugunruhe characterised in captive settings were generally timed similarly at both sites and coincided with actual migration of wild conspecifics. However, under constant conditions, Zugunruhe progressively drifted away from the calendar year, indicating a free-running rhythm. These studies thus demonstrated that migration is timed by an innate circannual clock (Gwinner 1986).

Circannual studies often investigated Zugunruhe as an integral part of the complete annual cycle, which in birds also includes periodic moult and reproductive activation and regression (King 1968; Gwinner 1986; Kumar et al. 2006). Research on captive migrants furthermore revealed recurring cycles of other processes linked to migration (King and Farner 1963; Dolnik and Blyumental 1967; Bairlein and Gwinner 1994). Many physiological and metabolic adjustments are carried out in step with migration (overview in Piersma and Van Gils 2011; McWilliams et al. 2022)). For example, to prepare for their formidable journeys, birds deposit fuel through temporary hyperphagia preceding migration (Piersma and Van Gils 2011). This hyperphagia can result in doubling of body mass, which is lost by the end of a migration season even in captivity. On a diel timescale, studies on Zugunruhe have provided evidence for circadian control of migratory restlessness and a range of related processes, for example a shift from anabolic physiology during the day to catabolic activity at night (Landys et al. 2004; McWilliams et al. 2022).

Our understanding of the navigation of migratory birds is also largely based on experimental approaches using Zugunruhe as a study tool. Gustav Kramer discovered that Zugunruhe behaviour is directed, i.e. caged migrating songbirds move in a direction corresponding to the migratory direction of free-flying conspecifics (Kramer 1949; Emlen and Emlen 1966). This solidified the perceived link between Zugunruhe and real migration and established orientation experiments as a tool for investigating the mechanisms and sensory pathways that underlie the birds’ navigational abilities.

A successful migration programme must closely integrate temporal and spatial aspects, so that appropriate directions are taken at the right time. It was therefore proposed that the integration of an innate sense of direction with an endogenous (i.e. innate) sense of time could function as a clock and compass mechanism, also referred to as “vector navigation”, whereby the angle of the vector represents direction, and the length represents time (Kramer 1957; Gwinner 1996a). Experiments involving naive birds using circular “Emlen” funnels indeed demonstrated an innate directional preference (Helbig 1996). Moreover, preferred direction changed between and within migration seasons also under constant, circannual conditions (Gwinner and Wiltschko 1980). A clock and compass mechanism could thus guide naive birds before learning and experience could inform navigational processes (Jenni and Schaub 2003; Perdeck 1958). Jointly, captivity experiments thus demonstrated that migration is based on a comprehensive, genetically hard-wired spatio-temporal migration programme.

Comparative studies revealed finely differentiated migration programmes between closely related taxa or sometimes even different populations of the same species, which further supports inheritance (see below). Moreover, these programmes orchestrate interactions with environmental cues (Gwinner 1986, 1996b; Pittendrigh 1993) through reaction norms that regulate phenotypic plasticity in response to specific environmental factors (van Noordwijk et al. 2006). Migrants show additional flexibility, for example by learning or social transmission (Madsen et al. 2023; Newton 2008; Åkesson and Helm 2020). Differences in relative rigidity vs. flexibility of migration, and in the response to specific environmental factors, may contribute to differentiation of migration behaviour between closely related taxa (van Noordwijk et al. 2006).

The persistence of local differences in temporal and spatial programming inspired evolutionary biologists to use Zugunruhe for investigating the genetic regulation of migration. Using the Blackcap (*Sylvia atricapilla*) as a study species, Berthold and colleagues showed strong genetic signal in key components of Zugunruhe behaviour (Berthold 1988a; Berthold et al. 1992). These studies involved decades-long selective and cross-breeding experiments of Blackcaps from populations with a variety of migratory phenotypes: from resident to long-distance migrant, and across migratory divides (i.e. closely neighbouring populations with different migratory directions). Behavioural phenotypes exhibited by F1 hybrid offspring (e.g., Zugunruhe propensity, timing, direction, and strength) were intermediate relative to parental populations (Berthold 1988a; Helbig 1996). One key finding was substantial microevolutionary potential of migration-related behaviour, based on the high heritability and strong response to artificial selection of Zugunruhe intensity levels (Berthold et al. 1992; Pulido and Berthold 2010).

#### Caveats and insights from field and captivity studies

The use of Zugunruhe as a proxy for migration behaviour has also limitations, and consequently, some caution is needed when drawing conclusions. In particular, correspondence between laboratory and field behaviour is not always clear. For example, bird populations with a sedentary phenotype in the wild may exhibit seasonal migratory restlessness in a caged setting, even in cases when they have been isolated from currently migratory populations for millions of years (Berthold 1988b; Mewaldt et al. 1968; Helm and Gwinner 2006). The timing of restlessness also does not always coincide perfectly with that of wild conspecifics. Caged migrants may exhibit Zugunruhe well beyond the end of actual migration, especially in the summer when bouts of nocturnal activity may continue until the onset of post-nuptial moult, despite reproductive activation ((Gwinner and Czeschlik 1978) Fig. S1).

Different explanations for such discrepancies have been discussed. Thus, deviant Zugunruhe patterns could be based on migration programmes whose expression is modified in response to deficient or misleading laboratory conditions (Gwinner and Czeschlik 1978). In the wild, avian migrants adjust the onset, progress and termination of their actual journeys in response to environmental factors (Jenni and Schaub 2003). For example, changing geomagnetic cues may indicate progress towards a destination (Fransson et al. 2001; Bulte et al. 2017), and availability of a territory or a mate may signal arrival on the breeding grounds (Newton 2008). These conditions do not occur in captivity, and Zugunruhe could simply reflect a general, flexible time window during which actual migration can be triggered or inhibited by environmental conditions (Gwinner and Czeschlik 1978; Helms 1963).

Rapidly advancing tracking technology allows for more direct comparisons between behaviour in captivity and the wild. Year-round approximations of migration routes and phenology (i.e., seasonal timing) by light-logging geolocators can be obtained even from tiny songbirds such as the *Phylloscopus* warblers referred to above (Gwinner 1969; Tøttrup et al. 2018; Sokolovskis et al. 2023). Slightly larger birds, such as Northern Wheatear (*Oenanthe oenanthe*) or Tawny Pipit (*Anthus campestris*), can carry multisensor loggers with additional functions (accelerometry, air pressure and temperature sensors) that allow derivation of daily movements and even actograms of free-flying birds (Fig. 2b) (Bäckman et al. 2017; Rime et al. 2023; Briedis et al. 2020). Data so far indicate that within species, phases of Zugunruhe indeed set a window in the annual cycle during which wild birds migrate (Jarrett et al. 2021; Akesson et al. 2017). However, actograms of free-flying birds also reveal stark differences from those of captive birds (Fig. 2). Whereas Zugunruhe often persists for weeks or even months, wild birds only fly for few selected nights and thereafter rest and refuel.

Comparisons of wild and captive behaviour also suggest that some Zugunruhe patterns might mirror nocturnal activities of wild birds not directly linked to migration (Fig. S1). Diurnal birds also naturally engage in nocturnal activity for other reasons, such as European nightingales (*Luscinia megarhynchos*) carrying out nocturnal courtship and prospecting behaviours (Roth et al. 2009), and Reed warblers (*Acrocephalus scirpaceus*) performing homing flights and change of breeding sites at night (Mukhin et al. 2009). Juvenile Reed warblers displayed nocturnal flights in the wild well in advance of migration, presumably to form memory for navigation, and corresponding nocturnal restlessness in captivity (Mukhin et al. 2005). In late summer birds may undertake post-breeding movements, for example for dispersal or to reach moulting locations (Pillar et al. 2015; Vīgants et al. 2023).

In winter or during migratory stopover, birds re-initiate nocturnal flights in response to environmental factors such as feeding opportunities (Åkesson and Helm 2020). For example, visible body fat, a measure of fuel availability, predicted the nocturnal departure of wild migratory birds from a stopover site (Goymann et al. 2010). Effects of feeding conditions and body fat on nocturnality were confirmed by experimental studies. For example, in captive Garden warblers (*Sylvia borin*) food availability and fuel stores function as a switch that can initiate or terminate migratory restlessness during and outside migration seasons (Gwinner et al. 1988b). Temporary food reduction during autumn migration increased Zugunruhe, but upon ad libitum food provisioning the birds paused nocturnality until fat stores were replenished, and then resumed migratory restlessness. Food-related switches to nocturnality, however, did not modify the annual timing, neither in Garden warblers (Gwinner et al. 1988b) nor in Wood thrushes (*Hylocichla mustelina*) (Stanley et al. 2022). Yet, not all switches to nocturnality observed in captivity align with behaviour in the field. Circadian lability could also be due to factors associated with captivity such as acclimatization or lack of local cues that signal arrival at target locations (Fig. S1).

Nonetheless, studies that tracked birds after quantifying Zugunruhe parameters found an increasing probability of migration in individuals that exhibited increased levels of Zugunruhe (Eikenaar et al. 2014). Furthermore, the directional preference of birds in orientation cages corresponded well with their migratory direction as assessed by subsequent tracking (Thorup et al. 2011). Thus, overall, Zugunruhe-based methods continue to reveal important insights on timing, navigation and compass systems of birds (e.g. Zapka et al. 2009; Kishkinev et al. 2016; Brodbeck et al. 2023)).

### Mechanisms

Despite a demonstrated heritable component of migratory behaviour, the mechanisms underlying its timing are still poorly understood. It is, however, clear that both circadian and circannual timing systems are involved. First, migration timing on diel and annual timescales shows that both systems are integrated (Figs. 1, 2). Second, since photoperiodism plays a major role in the annual timing of migration, the circadian system is involved through time measurement and through providing photo-inducible phases (Appenroth et al. 2021; Foster and Kreitzman 2009). Third, it is possible that both timescales are partly linked through shared underlying regulatory components, e.g., genes for proteins involved in photic or metabolic pathways (Bossu et al. 2022). Below we describe main mechanistic features for circadian and circannual time-scales on behavioural, physiological and molecular levels.

#### Circadian time-keeping: behaviour

Circadian rhythms of birds are well characterized on a behavioural level (Cassone and Kumar 2022; Helm 2020). Diel activity patterns in songbirds are generally robust, with the great majority of species being diurnally active (Daan and Aschoff 1975). Rhythmicity persists under constant conditions with well-described response features of the free-running circadian period length (Aschoff 1979; Cassone 2014; Kumar et al. 2004). Avian circadian rhythms readily entrain to photic Zeitgebers (i.e., synchronizing cues), including to changes in spectral composition (Pohl 1999), but alternative entrainment e.g., through phased melatonin (Heigl and Gwinner 1994), food (Hau and Gwinner 1997) or social cues (Menaker and Eskin 1966), is also possible.

Diel rhythmicity is less robust in some other avian groups, for example anatids (ducks, geese and swans) that can also be active at night, and charadriiforms, whose activity patterns may reflect tidal rhythms and are flexibly adjusted during the breeding season (Helm et al. 2012; Bulla et al. 2016). Accordingly, some features of the circadian system differ starkly between taxa and ecological niches. For example, the circadian system of songbirds is generally strongly self-sustained, as illustrated by weakly resetting (type-1) phase responses of European starlings (*Sturnus vulgaris*; (King et al. 1997)). In contrast, Japanese quail (*Coturnix japonica*) had a weakly self-sustained circadian system, as exemplified by their strongly resetting (type-0) phase response curve (King et al. 1997). Nonetheless, even in migratory songbirds, diel behaviour can be labile, as detailed above.

Modified rhythmicity gives clues to the diel architecture of the circadian system. In songbirds, circadian rhythms consist of multiple components that can be experimentally dissociated. For example, starlings continued to show rhythmic feeding activity even under bright constant light, whereas their locomotor activity became arhythmic (Gänshirt et al. 1984). These findings were interpreted as arising from two endogenous circadian oscillators that independently control rhythms in locomotor activity and feeding. Similarly, testosterone in starlings induced splitting of the free-running activity rhythm into two components that continued to run with different period lengths, again indicative of multiple underlying oscillators (Gwinner 1974).

**Fig. 3:**
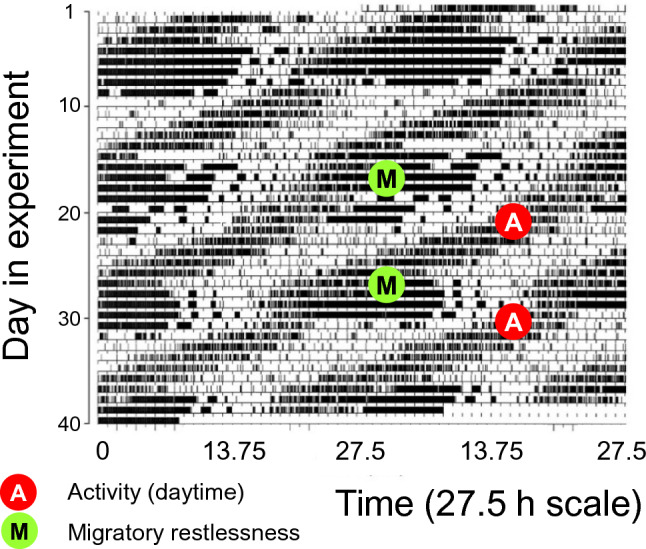
Dissociation of daytime activity (red A) and migratory restlessness (green M) of Garden warblers released from skeleton photoperiods to constant dim light. The intensely dark activity blocks represent migratory restlessness, whereas daytime activity is less solid. The rhythms of daytime activity and migratory restlessness cross due to their different period lengths. For better visibility, activity is plotted on a scale close to the periodicity of migratory restlessness (27.5 h); for details see (Bartell and Gwinner 2005). (Reproduced from Helm 2020 with permission, based on a figure kindly provided by Paul Bartell)

A multiple-oscillator system appears to also underlie the seasonal nocturnality of migrants. As illustrated for Zugunruhe in Fig. 2, an activity component appears to dissociate from diurnality to become migratory restlessness. Under entrained conditions, these two components tend to stabilize in antiphase to each other (Bartell and Gwinner 2005). However, under constant conditions or after weak entrainment, both activity components free-run with distinct period lengths in several study species. In all cases, the period length of migratory restlessness was longer, so that rhythms sometimes crossed (Fig. 3) (Bartell and Gwinner 2005; Kumar et al. 2006). Because of the demonstrated food-dependent switch to migratory restlessness, links of Zugunruhe to metabolic pathways have been proposed (Bartell and Gwinner 2005).

#### Circadian time-keeping: physiology

**Fig. 4:**
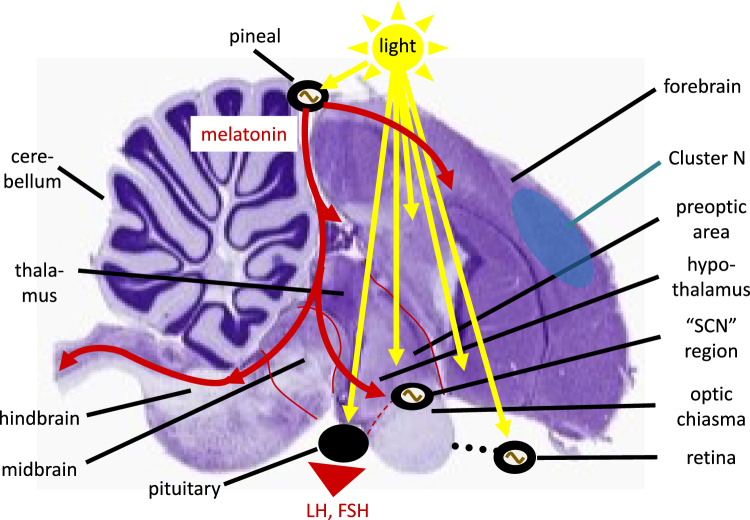
Timekeeping in brain and associated structures of a Great tit (*Parus major*). Black ellipses show approximated locations of structures central to circadian and annual timing (sine wave indicates pacemakers). Yellow arrows show light input pathways, red markings show major diel (melatonin) and annual (LH, Luteinizing Hormone; FSH, Follicle Stimulating Hormone) endocrine outputs; blue ellipse shows approximate location of Cluster N in a songbird which unlike Great tits migrates long distances. Thionin-stained sagittal section kindly provided by Davide Dominoni; figure slightly modified from (Helm 2020) and reproduced with permission

The core set-up of the avian circadian system is relatively well explored (Fig. 4; for overviews, see (Kumar et al. 2019; Cassone 2014; Helm 2020)). Briefly, in contrast to mammals, light input pathways to the circadian system in songbirds do not require ocular input. Based on conclusive experiments led by Mike Menaker, light input to the pineal gland, which in birds is located on top of the brain (Fig. 4), is sufficient to entrain the circadian clock (Menaker and Underwood 1976). However, light input can also reach the circadian system via deep-brain photoreceptors or indirectly via the retinohypothalamic tract to the SCN (suprachiasmatic nucleus). A third main light input pathway via the retina is important in columbids (pigeons) and galliforms (quail, chicken and landfowl; (Cassone et al. 2017)).

Remarkably and in further contrast to mammals, the avian pineal, SCN and retina can all function as pacemakers in their own right (Ma et al. 2019; Natesan et al. 2002). For example, the avian pineal is a miniature clock system that combines light receptors, self-sustained oscillators, and output pathways through production of melatonin (Helm 2020). The pacemaker structures communicate with each other through endocrine and neural pathways, and jointly function as a multiple photoreceptor–pacemaker system (Menaker and Underwood 1976). Through interactions, especially via melatonin and norepinephrine, they amplify each other and increase the circadian amplitude in what has been described as internal resonance through a neuroendocrine loop (Cassone et al. 2017). Yet the respective contributions of the different components differ between species. The multi-pacemaker system of birds with its many levels of regulation might facilitate the adjustment of diel rhythms, including rapid seasonal shifts to nocturnality during migration.

The hormone melatonin with its typical peak in darkness has a key systemic role in the circadian organization of most bird species (Helm et al. 2023), shown for example by its ability to synchronize circadian rhythms via phased oral intake (Heigl and Gwinner 1994). Yet variation in nocturnal melatonin amplitude indicates species-specific tuning. For example, whereas songbirds typically have pronounced melatonin rhythms, amplitudes are very low in charadriiforms (Helm et al. 2012). For migratory songbirds that adopt temporary nocturnality, links between melatonin and Zugunruhe have been demonstrated. During migration seasons, the melatonin amplitude is temporarily damped, and experimentally applied melatonin in turn reduces spring migratory restlessness (Fusani et al. 2013).

Other hormones widely implicated in circadian organization include glucocorticoids (Helfrich-Förster 2017; Caratti et al. 2018). Corticosterone, the main glucocorticoid in birds, also fluctuates on diel and annual timescales (Rich and Romero 2001), but its circadian functions are not well understood. In avian migrants, changes in the diel profile and amplitude of circulating corticosterone levels have been linked to migration (reviewed in (Bauer and Watts 2021)). Relationships between corticosterone and migration were not always consistent, but in several species, peaking corticosterone levels predicted migratory departure in wild birds and migratory restlessness in captive birds (Eikenaar et al. 2014). Furthermore, corticosterone was elevated when internal conditions (i.e., fuel stores) and environmental conditions (e.g., wind) were suitable for migration (Eikenaar et al. 2018). Thus, corticosterone may contribute to the fine-tuning of migration timing through mediating departure decisions based on external and internal cues (Eikenaar et al. 2018; Bauer and Watts 2021; Landys et al. 2004).

#### Circadian time-keeping: molecular mechanisms

Molecular mechanisms of circadian timing in birds are only partly explored (reviewed in Cassone 2014; Helm 2020; Kumar et al. 2019). Given the broadly conserved features of the circadian system across taxa, it is common to adopt characterisations of gene function that are heavily biased towards mammalian annotation (Bossu et al. 2022). Generally, where studied in detail, gene homologies, circadian expression dynamics, and structure of resulting proteins indicate that avian molecular functions are indeed often similar to those in mammals (e.g., core clock loop and photoperiodism; Nakane and Yoshimura 2010; Yasuo et al. 2003)).

**Fig. 5:**
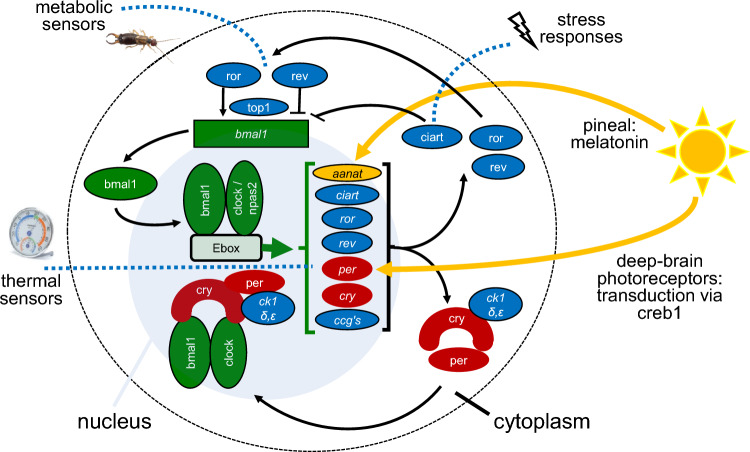
Schematic overview of molecular mechanisms of avian circadian timing. The figure shows the core clock loop with its positive arm indicated in green, and its negative arm in indicated in red. Accessory loops are indicated in blue, including the metabolic loop comprising ror and reverb, and a loop linking to stress responses involving *ciart*. Links to other factors affecting the timing of migration are indicated for photic input (in yellow) and for thermal sensors. For simplicity, only exemplary contributing molecules and some of their interactions are shown. Further pathways, such as immune pathways, are omitted. Genes are indicated by italics; for abbreviations, see Table S1 and text; ccg’s: clock-controlled genes

Briefly, the avian circadian system works through negative feed-back loops. These include transcription–translation feed-back loops of primary clock genes, whose gene products temporarily suppress their own transcription (mostly period genes, *per*; cryptochromes, *cry*; *bmal1* (arntl1), and *clock* / *npas2* (mop4) (name followed by aliases). These feed-back loops have a positive and a negative (suppressing) arm and are similar in birds and mammals (Fig. 5; for abbreviations, see Table S1) (Cassone and Kumar 2022). The positive arm (Fig. 5, green) involves the transcription factors bmal1 and clock / npas2. These transcription factors enter the nucleus and activate several genes whose promoters contain enhancer boxes (Ebox, a DNA response element) to which they bind. Period (*per2,3)* and cryptochrome genes (*cry1,2*,*4*) are activated by the bmal1-clock complex, initiating the negative arm of the core clock loop (Fig. 5, red). Their gene products form cry-per heterodimers in the cytoplasm which in the nucleus interfere with the bmal1-clock complex, thus repressing their own transcription. As in mammals, casein kinases (*ckδ,ε*) modulate the posttranslational degradation of per through phosphorylation and thereby influence the dynamics of the core loop. Further clock genes contribute sometimes alternatively, such as *dec* genes (BHLHE40, BHLHE41), or only in some tissues, such as *nfil3* (*e4bp4*)), which represses *per* activity in the Pars tuberalis of the anterior lobe of the pituitary gland (Yasuo et al. 2003; Natesan et al. 2002; Laine et al. 2019).

Like mammals, birds have further interlocked feed-back loops that integrate the circadian system with other important physiological pathways (Fig. 5). One main loop links the circadian and metabolic system through metabolic sensors ror (*rorα,β* (*NR1F1,2*)) and reverb (*nr1d1,2* (*reverbα,β*)), whose transcription is activated by the bmal1-clock complex. Rors have been characterized as lipid sensors and activate *bmal1* expression (Peek et al. 2012). Conversely, reverbs, which together with heme regulate gluconeogenesis and energy metabolism, suppress *bmal1* expression (Yin et al. 2007). Both sensors act competitively on *bmal1* via ror response elements (RORE) (Kumar et al. 2019; Peek et al. 2012). *Top1* (*topo1*) is thought to modulate the relative impact of *reverb* and *ror* action (Onishi and Kawano 2012). Another interlocking loop is thought to link the circadian system to stress responses, involving *ciart* (*chrono*) (Hatanaka and Takumi 2017) (Fig. 5). Additionally, interactions of the circadian system with glucocorticoid and mineralocorticoid receptors (*nr3c1 (gr*), *nr3c2* (*mr*)) suggest further links to stress response systems (Helfrich-Förster 2017). Ambient temperature additionally modulates circadian rhythms via transient receptor potential channels (TRP-channels) which are sensors, typically on the body surface, for ambient temperature and pain (Caro et al. 2013). and via heat-shock-factors (transcriptional regulators of genes for stress proteins) (Hirota and Fukada 2016; Reimúndez et al. 2023; Laine et al. 2019). These molecules have been associated with migration by changes in clock gene expression in central and peripheral tissues (Sur et al. 2020; Sharma et al. 2018).

The cycling and phasing of the clock gene variants can differ between species, between tissues within individuals and also between migratory and non-migratory phases within a species (Singh et al. 2015; Mishra et al. 2018; Renthlei et al. 2019; Horton et al. 2019). Epigenetic modifications might also play a role, as suggested by possible effects of methylation of *clock* on migration timing, but this area is poorly explored in studies of avian migration (Singh et al. 2019; Saino et al. 2017).

How the avian circadian system is entrained to or reset by light is also largely unclear (Fig. 5). Avian light input pathways use opsins that are expressed in circadian centres and widely in the brain and associated structures (OPNs1-5; Table S1), as well as the photopigments pinopsin (pineal) and vertebrate ancient opsin (VA; pre-optic area). Some of these photopigments may be specialized on photoperiodic input to annual time-keeping (Rios et al. 2019; Cassone 2014). It is possible that photic input to the circadian clock uses similar molecular pathways as in mammals (Meijer and Schwartz 2003). In the mammalian SCN, this involves activation of the mediator creb1 through its phosphorylation along MAPK signalling pathways within minutes after light exposure. Creb1 then binds to cAMP response elements (CRE) and thereby activates core clock and immediate early genes (e.g., *per, fos*; (Brenna et al. 2021; Mishra et al. 2018; Natesan et al. 2002)).

In birds, a further candidate pathway for light entrainment of the molecular clock is via melatonin (Cassone and Kumar 2022). The rhythmicity of melatonin biosynthesis derives from the enzyme aa-nat, whose activity is regulated by cry-per and bmal1-clock complexes and by the sympathetic nervous system (Chong et al. 2000; Klein et al. 1997; Natesan et al. 2002). Melatonin in turn increases *bmal1* transcription (Beker et al. 2019). Melatonin can be highly responsive to light, with rapid degradation after exposure (Klein et al. 1997), but also with induction shown in response to light of specific wavelengths (Ma et al. 2019). This pathway could be particularly relevant in birds, given melatonin’s role for internal resonance between avian pacemakers (Kumar et al. 2004; Cassone et al. 2017). Finally, some cryptochromes are (*cry4*), or may be (*cry2*), photosensitive in birds, with putative roles in magnetoreception and possible circadian links (Balay et al. 2020; Xu et al. 2021; Langebrake et al. 2023).

#### Annual time-keeping: behaviour

A circannual basis to migration programmes is documented in various species (Gwinner 1986; Karagicheva et al. 2018). However, demonstrating robust endogenous circannual rhythms, i.e., those that persist under constant conditions with nearly annual period length, is difficult because it requires maintaining birds individually for over a year indoors (Gwinner 1986). The permissive conditions, i.e., the light regimes under which a given species expresses circannual rhythms, vary widely among species. Some species only show cycles under a narrow range of photoperiods and otherwise get locked in one life-cycle stage, e.g., reproductive activation, others have weakly self-sustained circannual rhythms that require an environmental stimulus to complete a full cycle. Yet others have robust rhythms under a broad range of constant photoperiods including continuous dim light (e.g., Holberton and Able 1992; Wingfield 1993; Gwinner 1986). The degree of robustness and specifics of the permissive conditions are related to the migratory behaviour of a species. Generally, species with long-distance migrations express particularly robust circannual rhythms that persist under a wider range of photoperiods (Gwinner 1996b).

Studies of robust circannual rhythms give important cues to the underlying rhythmic organization. Rhythms can persist for over 10 years with period lengths that are mostly shorter than 1 year in songbirds (ca. 10 months), but longer than 1 year in waders (ca. 14 months (Karagicheva et al. 2018)). Circannual rhythms can be expressed from hatching, as shown for birds that hatched under constant photoperiod and developed full rhythmicity (Gwinner 1996b). Intriguingly, circannual studies showed that avian annual cycles are composed of modular processes that sometimes dissociate. For example, in some studies, moult continued to be rhythmic even if reproductive condition got locked in one state. Studies also showed that such distinct processes can cycle with different period lengths so that phase relationships progressively change (Karagicheva et al. 2016). In some songbirds, for example, postbreeding moult came to fully overlap with, or even precede, reproductive activation (Gwinner 1986).

Circannual rhythms of birds entrain readily to photoperiodic cycles. For example, starlings can be entrained to up to 12 cycles per year (Gwinner 1986). In some species, entrainment is possible even to low-amplitude photoperiodic cycles or to other photic cues, such as cycles in sunrise time or in light intensity under otherwise constant 12 h days (Gwinner and Scheuerlein 1998; Goymann et al. 2012). However, responses to the Zeitgeber are phase-specific, so that for example long-day stimuli in subjective autumn of high-latitude species delay the cycle, whereas the same stimuli in subjective spring advance it (Gwinner 1996b; Helm et al. 2009). Phase-specific responses, including unresponsive phases during the subjective winter, had been postulated on theoretical grounds to prevent migratory birds from initiating breeding on winter grounds during the austral summer (Gwinner 1996b; Hamner and Stocking 1970), and were subsequently experimentally demonstrated (Gwinner et al. 1988a).

**Fig. 6:**
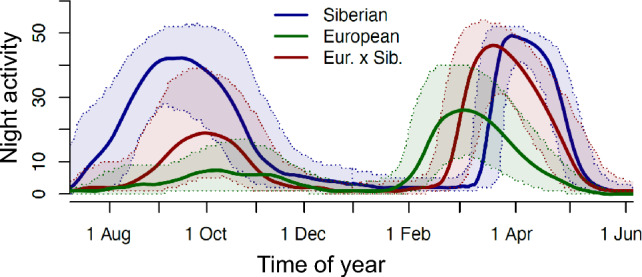
Migratory restlessness across the annual cycle of Siberian and European stonechats, as well as their F1-hybrids. Population differences observed in their natural habitat persisted in captivity, and cross-bred hybrids showed intermediate patterns. Activity levels are quantified as the number of nocturnally active ten-minute intervals of each individual; curves are medians with interquartile ranges; figure based on data from Van Doren et al. (2017)

While studies under constant conditions laid out the principles underlying migration programmes, studies under annually changing photoperiods are more directly applicable to natural behaviour. Common garden experiments under controlled conditions allow for some disentangling of genetic programmes and behavioural flexibility (van Noordwijk et al. 2006; Ketterson et al. 2015). In several species, differences in migration between closely related taxa that are observed in the field persisted in captivity. Figure 6 illustrates such differences for Stonechats from a long-distance Siberian population (*Saxicola maurus*) and a short distances population in Europe (*Saxicola torquata*) (Van Doren et al. 2017). Both taxa showed clear migratory restlessness across the annual cycle, but levels were much higher in Siberian than European stonechats. Both populations also differed in timing, whereby the Siberian stonechats initiated post-breeding migration earlier, and pre-breeding migration later in the year, compared to European stonechats. These differences closely mirror the behaviour of the two taxa in the field. Moreover, F1 hybrids of the populations that were bred in captivity showed intermediate patterns (Van Doren et al. 2017).

**Fig. 7:**
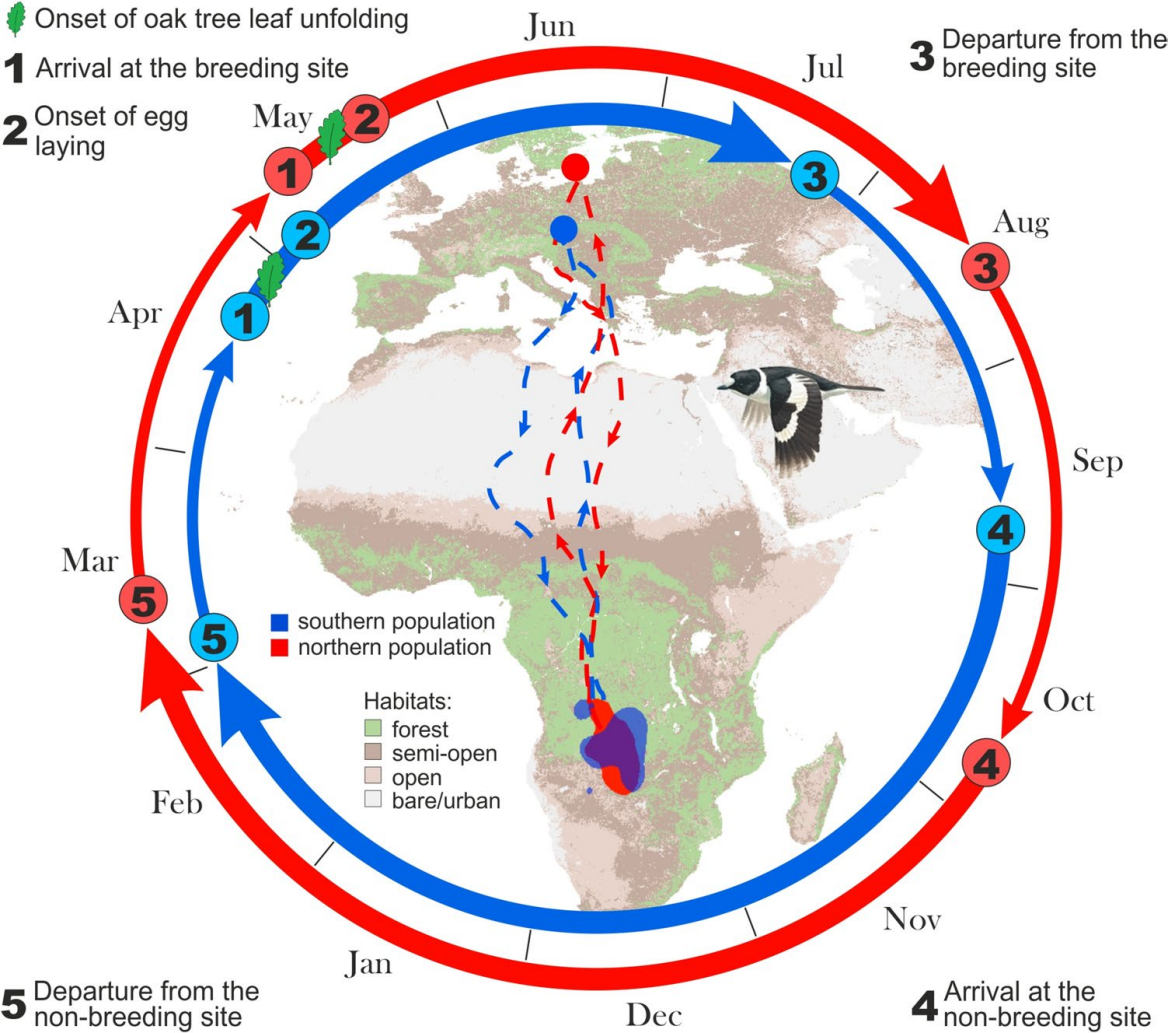
Migration programmes can differ between populations of the same species. Two populations of Collared flycatcher winter in the same area but their breeding sites differ in latitude; tracks are shown for northern, Swedish (red), and southern, Czech (blue) populations (numbers and letters indicate life-cycle events as indicated in the legend). Throughout the annual cycle, phenology of the southern population is advanced, roughly corresponding to earlier bud burst at the breeding sites. (Figure reproduced with permission from Briedis et al. (2016); see there for details)

Assessment of tracking data generally converged with captivity results in concluding that migration timing is partly genetically determined. Migration phenology within populations and even within individuals is often highly repeatable across years, whereas different populations often exhibit distinct timing patterns (Kürten et al. 2022; Franklin et al. 2022; Ketterson et al. 2015). Population-specific patterns are illustrated in Fig. 7 covering the full annual cycle for two populations of Collared flycatchers (*Ficedula albicollis*) (Briedis et al. 2016). The populations breed ca. 6° of latitude (ca. 800 km) apart, in Sweden and the Czech Republic, but their trans-equatorial wintering areas fully overlap. Nonetheless, seasonal timing of both populations differs year-around. As expected for their more northerly breeding sites, the Swedish birds arrived later in spring than the Czech birds, and this phase advance persisted throughout the year, albeit to varying magnitude. Since birds from both populations experienced identical daylengths during most of the winter, it is likely that they reset their annual cycle during migration or breeding.

**Fig. 8:**
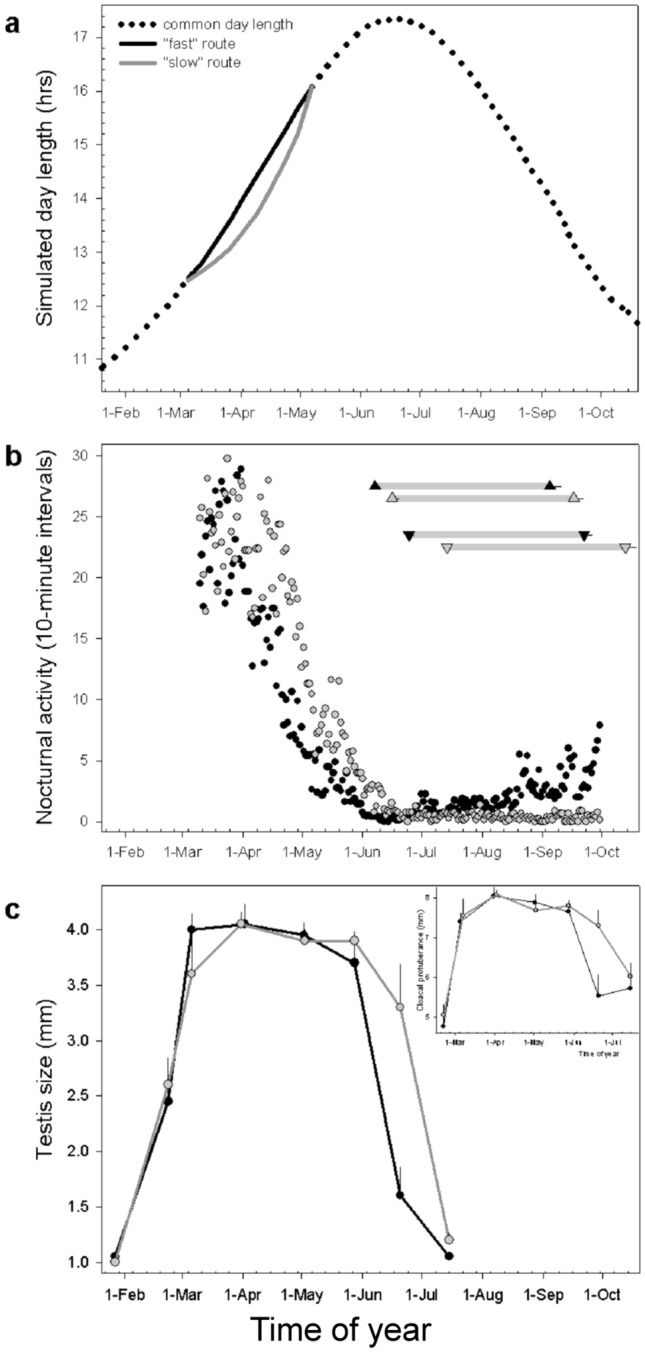
Sensitivity of European stonechats to spring daylength. (**a**) Two groups of males were kept under daylengths with varying speeds of spring daylength increase during a distinct time interval but were otherwise kept under identical conditions. The delayed spring increase, termed “slow” route, is shown by gray symbols, whereas the natural, “fast” route is indicated by black symbols. The slow-route birds delayed (**b**) the end of migratory restlessness, moult, and (**c**) reproductive regression, under a subsequently shared photoperiod. Moult is shown in the upper right corner within (**b**) by gray bars (upper pair: primary, lower pair: body moult); reproductive cycles in (**c**) show median testis size (main figure) and cloacal protuberance (inlay). (Reproduced with permission from Helm and Gwinner 2005)

Alternatively, or in addition, distinctly timed populations may have evolved specific photoperiodic responses (Singh et al. 2021; Helm et al. 2009). Experimental studies on stonechats detailed phase-specific use of photoperiodic information under naturally changing photoperiods (Fig. 8). Specifically, captive European stonechats were highly sensitive to subtle changes in daylength during the spring migration phase (Helm and Gwinner 2005). When the increase of spring daylength was temporarily slowed down during this sensitive window (simulating a slower route), the birds rapidly extended migratory restlessness. Although daylength of both groups was identical from the early breeding season onwards, the slow-route birds maintained a persistent delay of their annual cycle events. These “spring delayed” birds also delayed regression of the reproductive system, as well as initiation and completion of moult. The stonechats thus used spring photoperiod to entrain their circannual rhythm at a time when their reproductive system was already well developed (Fig. 8; (Helm and Gwinner 2005)). Comparative studies of Siberian and European stonechats furthermore showed that the populations differed in their response to simultaneously applied photoperiodic cues, presumably because they were in different phases of their annual cycles (Helm et al. 2009; Singh et al. 2021).

#### Annual time-keeping: physiological and molecular mechanisms

The anatomical structures that generate and orchestrate circannual rhythms are still poorly understood. In mammals, the Pars tuberalis has been described by Lincoln and colleagues as a circannual pacemaker that regulates rhythms of secretion of prolactin, and thereby cycles of pelage moult (Lincoln et al. 2006). This pacemaker involves interactions between local prolactin-secreting cells and timer cells that receive and convey the systemic, melatonin-based daylength signal. Based on these findings, Lincoln proposed more generally that circannual rhythms are generated through interactions of tissue-based, epigenetically modulated, pacemakers with coordinating systemic signals that integrate and convey timing cues (Lincoln 2019). In birds, the pituitary and nearby regions of the hypothalamus are also implicated in annual timing (Fig. 4). However, within-tissue circannual time-keeping has not been shown in birds, and unlike in mammals, melatonin plays no central role in avian annual organization (Cassone and Yoshimura 2022). Circannual rhythms in various physiological processes continued also in pineal-ectomized birds, even when diel rhythmicity was abolished (Kumar et al. 2019). Since circannual processes can dissociate within individuals, e.g., between reproductive and moult cycles, it seems plausible that Lincoln’s principal model also applies to birds (Lincoln 2019).

In birds, photoperiodic information is received directly by hypothalamic deep-brain photoreceptors (Fig. 4). Avian and mammalian photoperiodic pathways converge in thyroid activation. Stimulating photoperiods trigger cascading effects, involving a population of ependymal cells, tanycytes, that line the 3rd ventricle and synthesize compounds of the thyroid hormone and retinoic acid pathways (Yoshimura et al. 2003; Nakane and Yoshimura 2010; Kuenzel et al. 2015; Helfer et al. 2019). Thyroid signalling activates reproductive pathways via the hypothalamic–pituitary–gonadal (HPG)-axis, whereas for avian migration, evidence for a regulatory role of thyroid pathways is so far weak (Ramenofsky 2011; Pérez et al. 2016). Along the HPG-axis, hypothalamic neurons release gonadotropin-releasing hormone (GnRH) that stimulates the secretion of LH and FSH from the pituitary gland (Fig. 4), leading to production of gonadal hormones (Visser et al. 2010; Cassone and Yoshimura 2022; Chmura et al. 2022). In migratory garden warblers, however, changes in GnRH and downstream processes did not require photoperiodic stimulation. Instead, these processes occurred spontaneously under constant photoperiod at approximately the right time of year, revealing underlying circannual mechanisms (Bluhm et al. 1991).

Photoperiod affects annual cycles also via parallel signalling of the retinoic acid (vitamin A) pathway that links to nutrient-sensing, energy balance and immune pathways (Helfer et al. 2019). Recently, an important role of retinoids has been elaborated also in the brain, including for circadian light responses (Natesan et al. 2002), and for neurogenesis and neuroplasticity in the eyes, hippocampus and hypothalamus (Ransom et al. 2014). The availability of fuel, an important component of the decision of birds to initiate migratory flights, or, conversely, to build further energy reserves for example during stopover (Goymann et al. 2010), appears to be assessed in the hypothalamus (arcuate nucleus, or infundibular region) (Stevenson and Kumar 2017; Watts et al. 2018). Several hormones are involved in the regulation of fuel storage, for example through hyperphagia, in particular Neuropeptide Y (NPY). Annual-cycle timing is also sensitive to ambient temperature cues, which likely are transduced via TRP channels (McKechnie 2022; Caro et al. 2013). Temperature information is processed in the preoptic area of the hypothalamus, which is photoreceptive, and can also trigger thyroid action (Fig. 4). Temperature-sensitivity has been widely documented for avian migration, based on evidence from field and captivity studies (Sur et al. 2020; Chmura et al. 2022). Stress responses, regulated through the hypothalamus, can also modify annual timing (Wingfield 2012). Corticosterone levels of birds change over the annual cycle and have been implicated in the control of migration (Bauer and Watts 2021; Eikenaar et al. 2018; Landys et al. 2004). All these pathways are also modulated by, and interact with, the circadian system.

Still, the anatomical substrates that underlie and integrate the contributing pathways are far from clear. A key integration site appears to be the hypothalamus (Cassone and Yoshimura 2022; Chmura et al. 2022; Mishra et al. 2018; Watts et al. 2018). Stevenson and Kumar (Stevenson and Kumar 2017) speculated that the anatomical organization of migratory timing involves the circadian pacemakers, the medio-basal hypothalamus, the dorsomedial hypothalamic nucleus (DMH) and the adjacent infundibular region (IR, arcuate nucleus). In this model, the DMH, IR, and pre-optic area are central for integrating energy balance. The anatomical organization also includes a forebrain area involved in magnetic compass orientation whose activation correlates with Zugunruhe, Cluster N (Fig. 4) (Brodbeck et al. 2023). Jointly, these structures might form the substrate for the integrated spatio-temporal programme of migratory birds (Stevenson and Kumar 2017).

**Fig. 9:**
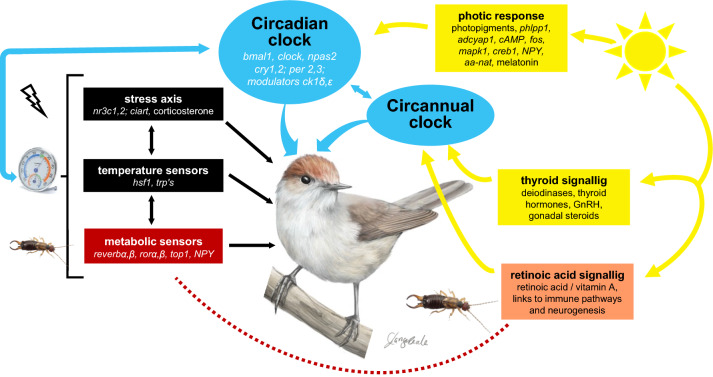
Pathways acting on annual timing of birds. Bird migration is shaped by complex interactions between the endogenous circadian and circannual clocks, light input, further environmental factors like stress, food and ambient temperature, and internal factors like nutrient supplies and metabolic state. Clock components are shown in blue, light input pathways in yellow, and metabolic pathways in red (retinoic acid signalling is in orange as it contributes to light input and metabolic pathways); further pathways are shown in black. For simplicity, only exemplary compounds of pathways, and interactions between pathways, are indicated, and some systems (especially immune pathways) are omitted. Genes are indicated by italics; for abbreviations, see Table S1 and text. Modified after (Helm et al. 2023). Inlay shows a Blackcap, drawn by Corinna Langebrake

Molecular details of annual timing mechanisms in birds remain largely unclear but are inferred through two approaches. First, knowledge of involved pathways is used to generate lists of candidates that have been functionally described or tested mainly in mammals. Variation in these candidate molecules is studied correlatively by comparing taxa that differ in migration or annual-cycle timing. They are also sometimes experimentally tested, e.g., by quantifying expression levels at contrasting time-points, such as during or outside migration seasons (Mishra et al. 2018; Singh et al. 2015). A second, untargeted, approach aims to identify genes or regulatory pathways de novo by comparing genetic variation (Single-Nucleotide Polymorphisms, SNPs) between populations exhibiting different migratory strategies (e.g., migrants *versus* residents, different phenologies or routes (Delmore Kira et al. 2016; Bossu et al. 2022; de Greef et al. 2023; Lundberg et al. 2017)). Interpreting results from untargeted approaches in a functional context can be facilitated by charactersing differential expression patterns in focal tissues at contrasting phases of the annual cycle (Boss et al. 2016; Johnston et al. 2016; Horton et al. 2019; Frias-Soler et al. 2020; Franchini et al. 2017). Genes that are differentially expressed form a rapidly growing list of candidates for deciphering the regulatory machinery underlying migration. These genes point to key signalling pathways, involving the metabolic, circadian and stress systems (overview in Fig. 9), as well as neurodevelopment, immune pathways, and memory and learning. A recent list has been compiled by (Bossu et al. 2022; Lugo Ramos et al. 2017).

Perhaps unsurprisingly given the many knobs and bolts of time-keeping systems, results from various bird systems were mostly mixed and identified different targets of selection. However, there was some convergence in molecules identified by candidate and untargeted approaches, involving the key pathways described above. Main examples include the core clock loop (*clock* and *npas2*), genes related to photic input pathways (e.g., *creb1*, *adcyap1*, *phlpp1*), and genes associated with nutritional sensors (e.g., *top1*) ( (Bossu et al. 2022; Le Clercq et al. 2023; Lugo Ramos et al. 2017) and review therein).

### Challenges and responses in a changing world

Global environments are changing at ever faster rates, and many changes have a strong temporal component. Avian migrants are particularly susceptible to such changes, because they depend on the conditions at multiple places that are separated by time and space. Successful migration requires integrated interaction with environments over several spatio-temporal stages. Each stage is sensitive to changes that may be poorly correlated over space and time, so that simple adjustments of migration may often not suffice (Vickery et al. 2023; Newton 2008). Migratory species are thus facing major threats and are broadly declining (Wilcove 2008; Bairlein 2016). For example, long-distance migrants show limited scope for advancing phenology and suffer particularly negative population trends (Howard et al. 2020; Usui et al. 2017; Vickery et al. 2023; Youngflesh et al. 2021). In a demographic analysis of long-distance migrants, a close association between warmer springs on the Czech breeding grounds and reduced breeding productivity explained a large proportion of inter-annual demographic variation (Telenský et al. 2020). An association between phenology and population trends was shown in detail for one species, the Pied flycatcher (*Ficedula hypoleuca*) (see below, (Both et al. 2006)).

Above, we have shown that findings from studies in the wild, captive experiments and molecular analyses all converged on identifying an inherited basis to migration. Migratory traits that strongly rely on innate genetic programmes may thus be limited in flexibility, requiring evolutionary adjustments for tracking environmental change. Such change can initially occur rapidly through adaptive evolution (Bonnet et al. 2022), whereby standing genetic variation provides a basis for adjustment through natural selection (Helm et al. 2019; Van Doren et al. 2021; Delmore et al. 2020). Conversely, evolutionary adjustments that require de novo mutations might be much slower. We also showed ample phenotypic plasticity that is partly regulated through migration programmes, for example in response to food availability or geomagnetic cues (Åkesson and Helm 2020). Such plasticity may facilitate direct responses to changing environments, up to a point when underlying reaction norms themselves need to change (van Noordwijk et al. 2006; Nussey et al. 2005). Additional behavioural flexibility, for example via social transmission, can also modify speed of adjustment (e.g., (Madsen et al. 2023)).

Overall, migration strategies display an astounding spectrum of variation. At one extreme is high conservatism, for example in migratory populations that have fixed schedules (Both and Visser 2001) or adhere to evolved routes, even when they imply enormous detours (Kürten et al. 2022; Bairlein et al. 2012). A striking example are Northern wheatear populations that breed in Alaska and migrate more than 14,000 km each way to sub-Saharan winter quarters in Africa, rather than overwintering in the neotropics (Bairlein et al. 2012). At the other extreme are fast and sometimes fundamental adjustments of migration, for example by swallows and geese (Areta et al. 2021; Madsen et al. 2023). This diversity may be partly due to different behavioural contexts and environmental sensitivities (Newton 2008; Youngflesh et al. 2021; Hardesty-Moore et al. 2018), and partly due to evolutionary constraints and genomic architecture (Taylor and Friesen 2017; Lundberg et al. 2023).

Historically, migrations have shown major evolutionary changes, for example in step with glaciation cycles that starkly altered spatio-temporal conditions (Thorup et al. 2021). Thus, whereas the machinery underlying main migration features, such as timing and navigation, is probably ancient, many current forms of migration have appeared since the last glacial maximum (Liedvogel et al. 2011; Rappole et al. 2003). Over evolutionary time, adjustments of ancient features have thereby facilitated a wide diversity of bird migrations, but the current rate of environmental change may push the dynamic features of migration programmes to the limit (Radchuk et al. 2019). Below, we summarize factors that contribute to altered or disrupted timing, either on their own or in interaction.

#### Environmental changes affect timing

A primary challenge for organisms is global warming, which progresses at ever faster rate and affects timing of wild organisms (IPCC 2023). Direct temporal effects include altered environmental seasonality and phenology (e.g., modified temperature and precipitation patterns). These effects can be amplified by different phenological responses among species and trophic levels, and can be complex due to spatial heterogeneity (e.g., different regions warm at different rates) and temporal heterogeneity (e.g., warming is stronger in some parts of the year) (Thackeray et al. 2010; IPCC 2023). The increasing variation in climate, such as intensity and frequency of temperature and precipitation extremes, adds further risks (Ummenhofer and Meehl 2017). For example, organisms that steadily advance spring phenology may fall victim to sporadic severe cold weather events (Brown and Bomberger Brown 2000; Shipley et al. 2020); for a tree example, see Fig. S2). These challenges are exacerbated for migratory birds that need to time their journeys to predicted conditions at multiple places across the annual cycle (Zurell et al. 2018). However, global warming currently also opens new opportunities, such as milder winters that allow birds to remain closer to the breeding grounds. Shortened migration routes in turn enable earlier spring return and greater predictability of conditions on the breeding grounds (Van Doren et al. 2021; Youngflesh et al. 2021; Visser et al. 2009).

A second temporal challenge to migratory birds arising through environmental change is artificial light at night (ALAN). Light pollution increases globally at ever faster rates (Kyba et al. 2017). It can have severe consequences for timing, orientation and navigation capabilities, of nocturnal migrant species. ALAN can affect migrants during breeding, at non-breeding sites and *en route* (Cabrera-Cruz et al. 2018). While the greatest immediate risk to birds may be disrupted orientation, the exquisite light sensitivity of their circadian and circannual systems also provides pathways for mistiming and disruption of migration programmes (Kumar et al. 2021). ALAN-induced migration phenology changes have recently been reported also in wild birds (Bani Assadi et al. 2022; Smith et al. 2021).

Land-use changes can also have temporal dimensions and may thereby alter the fitness consequences of inherited migration programmes. Examples are landfills which offer food year-round and thereby counteract characteristics of migration of White storks (*Ciconia ciconia*) (Flack et al. 2016) and increased breeding opportunities which were suspected to invert the annual cycle of swallows (see below (Helm and Muheim 2021)). Similarly, extending the size of barriers crossed on migration such as the Sahara desert is likely to slow migration due to the need for additional stop-overs (Goymann et al. 2010). Land-use change can, however, also open new opportunities, e.g., by creating novel breeding range for migratory species (Winkler et al. 2017; Areta et al. 2021).

Finally, various other forms of pollution (e.g. chemical, noise) may have implications for timing of migratory birds, but we currently lack detailed mechanistic knowledge for proper assessment. Delayed timing of migrants has been reported to result from neonicotinoid exposure, which in turn caused reduced fuelling and possibly disorientation (Eng et al. 2019). Impairment of migration programmes is also possible as neonicotinoids can disrupt circadian rhythms by interfering with nicotinic acetylcholine receptor signalling (Tasman et al. 2021). Birds are also exposed to particulate matter (PM2.5), which in mice causes circadian disruption, but no corresponding data exist for migratory birds (Palanivel et al. 2020).

Below, we review some of the observed responses in greater detail, with a focus on global warming. This overview is necessarily biased towards species that show responses that can be plausibly linked to environmental change, such as adjustment of migration route or timing (Radchuk et al. 2019). Species with limited response potential may only show negative population trends, while being most vulnerable to environmental change (Telenský et al. 2020).

#### Phenology changes due to global warming

Global warming, and associated changing environmental seasonality, have reportedly modified the timing of all avian life-cycle stages, including breeding, moult and migration (Hanmer et al. 2022; Lameris et al. 2018; Visser et al. 2004; Horton et al. 2020; Bussière et al. 2015; Jenni and Kéry 2003). These responses have been best documented for reproductive timing, where the rapidly advancing environmental phenology was nonetheless difficult for birds to match. Birds bred earlier, but their advancement was insufficient for tracking plant and invertebrate phenology, leading to mismatches with potentially detrimental consequences such as reduced breeding productivity (Visser et al. 2004; Samplonius et al. 2021; Radchuk et al. 2019).

While such mismatches affected even resident species, avian migrants were hypothesized to suffer greater mismatches given their strong programming. Support for this idea came from data on Pied flycatchers whose lay dates were constrained by arrival date (Both and Visser 2001). Since birds hardly advanced arrival date, they advanced lay date only by reducing the interval between arrival and laying, a short phase that migratory birds usually use to build breeding resources. Both and Visser showed that compared to ecologically similar resident species, Pied flycatchers progressively fell behind schedule. They also demonstrated that in areas where spring had advanced the most, flycatcher population trends were particularly negative (Both et al. 2006). Extrapolating these findings from a single species is speculative since various factors can affect vulnerability of a species (Hardesty-Moore et al. 2018). However, several lines of argument support a broader pattern. Thus, most long-distance migrants show limited advancement of spring activities and heightened population declines (Vickery et al. 2023; Howard et al. 2020; Usui et al. 2017; Youngflesh et al. 2021). Furthermore, warmer springs have been reported to correlate with low breeding productivity of long-distance migrants (Telenský et al. 2020), and in several avian species, a strong selection gradient favouring early breeding has been shown (Radchuk et al. 2019). Still, several studies suggest that in some species migration programmes have recently evolved (Able and Belthoff 1998; Delmore et al. 2020), including in aspects of timing (Bearhop et al. 2005; Moiron et al. 2023; Brown and Bomberger Brown 2000). These changes support the captivity evidence of high microevolutionary potential of migration-related behaviour.

**Fig. 10:**
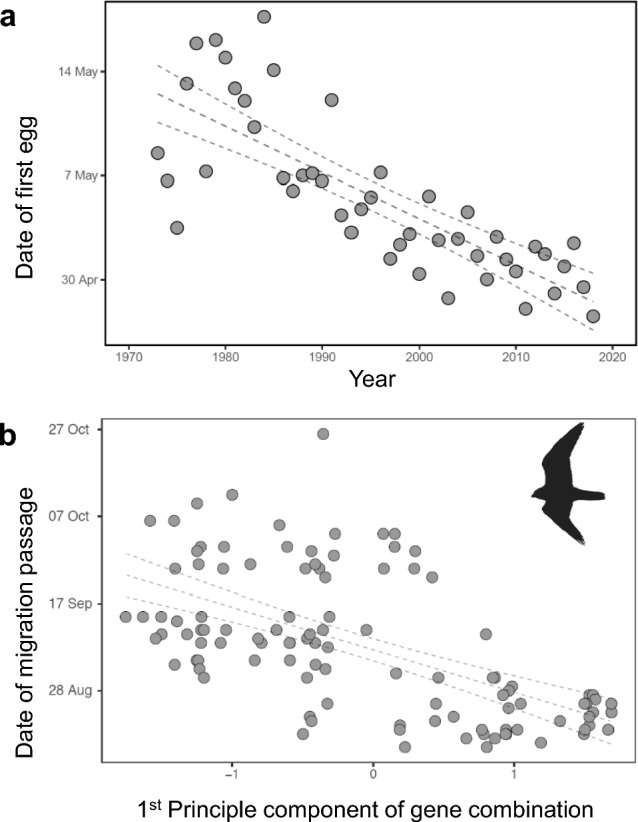
Response potential to changing phenology. (**a**) Many bird species advance their spring activities, here shown by the change in lay date of Pied flycatchers, which likely occurred through genetic change (data from (Helm et al. 2019)). (**b**) In American kestrels, intraspecific variation in phenology, here of autumn migration, can be partly explained by variants in a small number of genes, including those linked to clocks. (Data from Bossu et al. 2022)

A recent study on Pied flycatchers has linked captivity evidence of a migration programme with the birds’ phenology in the wild (Helm et al. 2019). Migration programmes of flycatchers in full annual-cycle context had been studied in captivity by Gwinner and colleagues in the 1980s (Gwinner 1989, 1996b). Since then, lay dates of free-living flycatchers had advanced considerably, including in southern Germany from where the captive birds originated, and where citizen scientists have continuously monitored flycatchers since the 1970s (Fig. 10; Both and Visser 2001; Helm et al. 2019)). Hence, a replication of the original captive study offered a unique opportunity to test whether changes in the wild were driven by modifications in the migration programme as studied in captivity. In a common garden experiment through time, nestlings from the same natal sites as in 1981 were collected in 2002, hand-raised, and their annual cycles studied under conditions that closely mimicked the original experiment (Helm et al. 2019).

The study showed that over 21 years, the flycatchers had indeed modified annual-cycle timing in captivity, but selectively so. Autumn and early winter activities were slightly delayed or unchanged, in line with evidence for far less systematic changes of migration phenology in autumn compared to spring (Jenni and Kéry 2003). In contrast, the flycatchers’ activities in late winter and spring were substantially advanced, as predicted based on field evidence for earlier laying. Spring activities in captive birds were advanced by ca. 9 days, whereas free-living conspecifics had advanced laying over the same interval by ca. 11 days (Fig. 10 (Helm et al. 2019)). The data suggest that a large part of the flycatchers’ spring advance was due to changes in the migration programme. The study also indicates that the birds can separately modify specific phases of the annual cycle, as opposed to phase-shifting the entire cycle (Tomotani et al. 2018). Such an ability, which fits findings of modular organization of avian annual cycles, could facilitate adjustments to space- and time-specific changes in climate (IPCC 2023).

The magnitude of inferred evolutionary change in flycatchers (9 days over 21 years) is high, also compared to data from some other species (Moiron et al. 2023). A possible explanation is the potential of timing to function as a “magic trait” (i.e., a trait that also causes assortative mating Taylor and Friesen 2017; Ketterson et al. 2015)), and thereby accelerate evolutionary change (Bearhop et al. 2005). Such rapid change would require substantial standing genetic variation for timing. High standing variation has recently been demonstrated in at least two species, American kestrel (*Falco sparverius* (Bossu et al. 2022) and Purple martin (*Progne subis*; (de Greef et al. 2023)), where variation in genes explained 33% and 74% of phenotypic variation, respectively. Figure 10 shows that focal genes in kestrels predicted phenological differences of similar magnitude as the spring advancement of flycatchers. Populations with such high levels of variation in timing are expected to be relatively robust in face of environmental changes (Bossu et al. 2022).

#### Altered spatio-temporal migration features

Alternatively, or in addition to, phenological adjustment, migratory birds respond to changes in seasonality and other environmental factors at breeding and non-breeding sites by spatial adjustments. Thus, poleward shifts of breeding and wintering ranges, as well as altered routes, have all been reported. Such spatial arrangements can have pronounced effects on timing. For example, shortened routes enabled by milder winters require less time for migration and may thereby advance subsequent life-cycle stages (Bearhop et al. 2005). Conversely, poleward shifts of breeding ranges but static wintering ranges are extending migration routes, with expected costs in time and energy (Gómez et al. 2021; Zurell et al. 2018). From a perspective of chronobiology, spatial re-arrangements are also expected to interact with timing mechanisms (Huffeldt 2020). Latitudinal shifts of breeding and non-breeding ranges can increase or decrease daylength experienced by birds at a given time of year depending on their location (Sockman and Hurlbert 2020). How birds will respond to such changes will depend on their phase-specific sensitivity to photoperiod, and is thus difficult to generalize (Fig. 8; Helm et al. 2009; Gwinner 1996b)).

Among the most extreme spatio-temporal shifts of migration were trans-hemispheric inversions. Apparently facilitated by landuse change and novel anthropogenic nesting opportunities, some individuals of the North American-breeding swallow species started breeding in their South American non-breeding range (Winkler et al. 2017; Areta et al. 2021). Remarkably, the birds kept their annual cycle intact and appeared to achieve the complete reversal in time and direction solely via re-entrainment (Helm and Muheim 2021). Rather than becoming resident, they continued their migrations, but with reversed direction, to now travel northward after breeding (Winkler et al. 2017; Areta et al. 2021). Modelling indicated that the migration programme can fully explain the birds’ spatial behaviour without need for genetic change (Fig. S3). On their North–South migratory route, with a magnetic inclination compass that is blind to polarity, hemisphere-switching swallows following the migration programme would continue to navigate between breeding and non-breeding grounds. All that is required is for the swallows to stay long enough at the non-breeding grounds to entrain their migration programme to the southern hemisphere (Helm and Muheim 2021). Being gregarious birds, it is possible that social transmission from conspecifics and closely related species aided the process. Social transmission was recently proposed to be the key driver of novel migration behaviour in another gregarious species, the Pink-footed goose (*Anser brachyrhynchus*) (Madsen et al. 2023).

Large-scale shifts in migration behaviour have also been observed across longitudes. In several central Asian-breeding species, some individuals are newly spending the non-breeding phase in central Europe, rather than at their traditional wintering grounds in Southeast Asia (Dufour et al. 2022, 2021). For example, the formerly vagrant Richard’s pipit (*Anthus richardi*) is now a regular winter visitor to France, as evidenced by inter-annual return of marked individuals, and by tracks of birds that bred in Siberia between winter visits to Europe (Dufour et al. 2021). For Richard’s pipit, this new migration route, putatively enabled by mild winters, almost doubles the migration distance and shifts migration direction from south to west. Pipits using the new route thus experience photoperiods similar to those of the breeding grounds around the year. Compared to the original southeast Asian winter grounds, they experience shorter daylength in winter, but longer, and potentially stimulating daylength during the pre-breeding phase after the equinox (Fig. 8; (Dufour et al. 2021)). Similar trends are observed in another Asian-breeding songbird, the Yellow‑browed warbler (*Phylloscopus inornatus*; (Dufour et al. 2022)). However, for both species there is insufficient information to assess whether genetic change is involved in altered migration, and whether the birds’ phenology has also shifted.

**Fig. 11:**
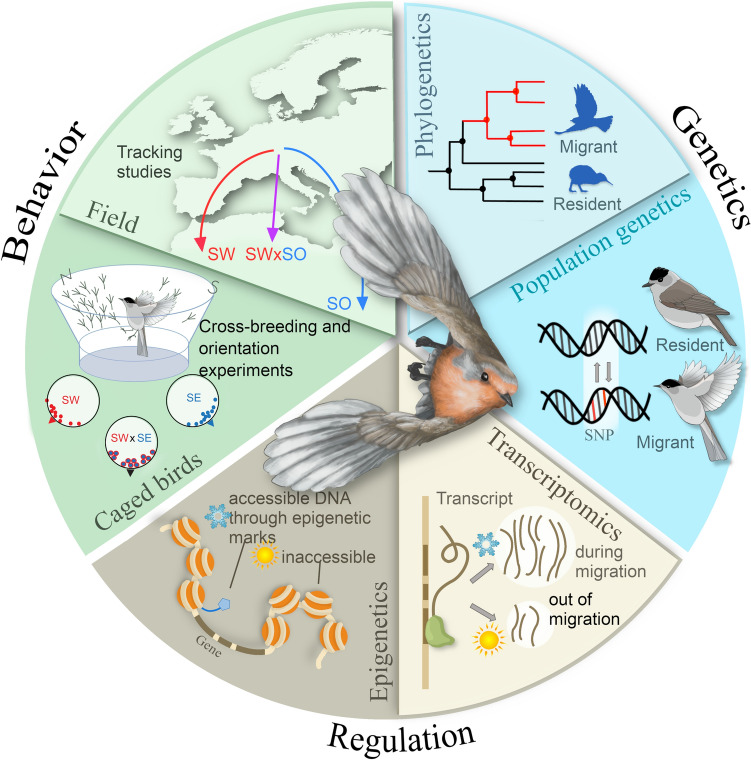
Overview of an integrative approach to studying avian seasonal migration. Behavioural (green), genetic (blue), and molecular (brown) methods can jointly reveal the mechanisms underlying the spatio-temporal migration program. Behavioural research is exemplified by comparing migration tracks in the wild and directional preference in Emlen cages between populations of Blackcaps that migrate southwesterly (SW) or southeasterly (SE). Genetics approaches compare genomes to identify ancestral states and candidate genes using broad phylogenetic scales and detailed comparisons, here between migratory or resident Blackcap populations. Molecular methods detail regulatory processes by seasonal contrasts for example in gene expression or epigenetic modulation. (Figure from Langebrake et al. 2021 under creative commons license)

Many species have shifted spatio-temporal migration features at smaller scales (Zurell et al. 2018). A textbook example that involved genetic change comes from Blackcaps, a species with a well-described migration programme (Berthold et al. 1992; Pulido and Berthold 2010). Central European-breeding populations used to spend the non-breeding season in the Iberian region, southwesterly of their breeding grounds. Since the 1960s, these birds increasingly spend their winters on the British Isles, westerly of the breeding grounds, probably due to combined effects of a milder climate and human food provisioning (Delmore et al. 2020; Van Doren et al. 2021; Bearhop et al. 2005). To test whether this new migratory divide had a genetic basis, Iberian and British-wintering Blackcaps were kept under common-garden conditions and tested in Emlen funnels for autumnal directional preference (Fig. 11). Those wintering in Spain showed southwesterly preferences, whereas those wintering in Britain oriented westerly. Remarkably, their naive, captivity-bred offspring mirrored the parental preferences (Berthold et al. 1992; Helbig 1996). A follow-up study suggested that British-wintering Blackcaps returned earlier to the breeding grounds and mated assortatively (Bearhop et al. 2005). Since earlier breeding birds usually have higher reproductive success, behavioural mechanisms have apparently accelerated genetic change in migration. At present, the genetic basis underlying a broad range of migratory phenotypes in Blackcaps is being unravelled (Fig. 11). First results indicate that several migration features map to a few genomic regions, which differ from those described in other species. However, possibly associated candidate genes that may be major regulators of migration include metabolic and circadian transcription factors, such as NPY (Watts et al. 2018)) and several clock genes (Table S1; Delmore et al. 2020; Mishra et al. 2018)). These data add to the emerging picture that variation in migration features can be achieved in multiple ways but often on converging physiological pathways.

## Conclusions and outlook

For over a century, the daunting ability of avian migrants to navigate across time and space has fascinated chronobiologists, ecologists and behavioural biologists alike. Research on captive and free-living birds so far has provided intriguing answers, but how long-distance migrants know when and where to travel is still only partly resolved. Key questions include several the enigmas. It is still unclear how long-term rhythms underlying migration, on the scale of a year, are generated, and how they interact with circadian time-keeping. It is also unclear how the many processes revolving around migration and the remaining activities of the annual cycle are integrated, so that they all occur at suitable times and locations. During their migrations and over the timescale of a year, birds are faced with a wide range of environmental uncertainty. Migration programmes must offer flexibility for birds to accommodate environmental fluctuations, while canalizing behaviour so that target areas are reliably reached at the right time. Furthermore, migrations are often tailored to the specific ecologies of a population, implying that genetic, epigenetic and experiential processes must be in place that finely adjust migration programmes. Many adjustments will be achieved through molecular processes along the intertwined pathways that orchestrate avian migration. At present, we are only beginning to perceive the scope of physiological processes involved in successful migration.

A better understanding of migration at physiological, behavioural and evolutionary levels is urgently needed. Migrants do not only perform some of the most fascinating behaviours, they are also under particular pressure in our rapidly changing world. Without a deeper understanding of underlying mechanisms, we have no explanations for widely disparate responses of birds to environmental change. We cannot explain why some species, such as Northern wheatears, choose conservative routes at the cost of enormous detours, and others, such as Richard’s pipits, rapidly develop completely novel migration programmes. Thus, we have no understanding of which species can succeed in the face of environmental change, and which species may decline towards extinction. Improving the knowledge basis will require integration on several levels (Fig. 11).

First, researchers with detailed physiological and molecular knowledge and those that study behaviour and ecology of free-living birds must reconnect. Genomic analyses of birds with known migratory phenotype, for example, produce lists of candidate genes, whose interpretation is beyond the capacity of most researchers that work in the wild. All too often, analyses focus on individual candidate genes, whose effects may be small and sometimes redundant within extensive functional networks. Moving towards network-based approaches will require substantial expertise. Furthermore, epigenetic modification has so far hardly been considered in migration studies, presumably due to missing expertise and tools. Chronobiologists, who by definition work on cross-cutting themes, are well-positioned to provide relevant expertise and facilitate future break-throughs in migration biology.

Second, new technologies, such as advanced tracking methods that yield actograms, or radar observations, allow for more detailed knowledge of timing and environmental responsiveness in the wild. Ideally, these methods should be paired with collection of genetic information and progressively also apply experimental approaches. Experimental approaches will be necessary to move from correlative to causal evidence. Next to experimentation in the field, captivity studies under controlled conditions remain crucial for a mechanistic understanding.

Third, long-term studies of free-living birds are key for deeper insights, given large-scale environmental fluctuations encountered by migratory birds. For example, changes in timing become evident only over long time spans and in standardised data series. If unified approaches comprising field and molecular aspects are applied, individual variation within well-described systems can give important cues to physiological, ecological and evolutionary mechanisms that shape avian migration. A growing, comparative basis of such systems, if thoroughly reviewed and integrated, will shed light on regulatory mechanisms that enable, or conversely constrain, appropriate adjustment of migration programmes in a changing world.

### Supplementary Information

Below is the link to the electronic supplementary material.

Supplementary file1 (PDF 861 KB)
