# Supplementary material for: Avian migration clocks in a changing world
Source: J Comp Physiol A Neuroethol Sens Neural Behav Physiol. 2024 Feb 2;210(4):691–716. doi: 10.1007/s00359-023-01688-w (PMC11226503; doi:10.1007/s00359-023-01688-w)
Supplement: Supplementary file 1 — Supplementary file1 (PDF 861 KB) [file 359_2023_1688_MOESM1_ESM.pdf]

Supplementary Information

Avian Migration Clocks in a Changing World

Barbara Helm & Miriam Liedvogel

Swiss Ornithological Institute, Bird Migration Unit, Seerose 1, CH-6204 Sempach, Schweiz; barbara.helm@vogelwarte.ch
Institute of Avian Research, An der Vogelwarte 21, 26386 Wilhelmshaven, Germany; miriam.liedvogel@ifv-vogelwarte.de

Contribution for edited volume: A clock for all seasons (Charlotte Förster and Dirk Rieger, editors; *Journal of Comparative Physiology A*)

Supplementary figures

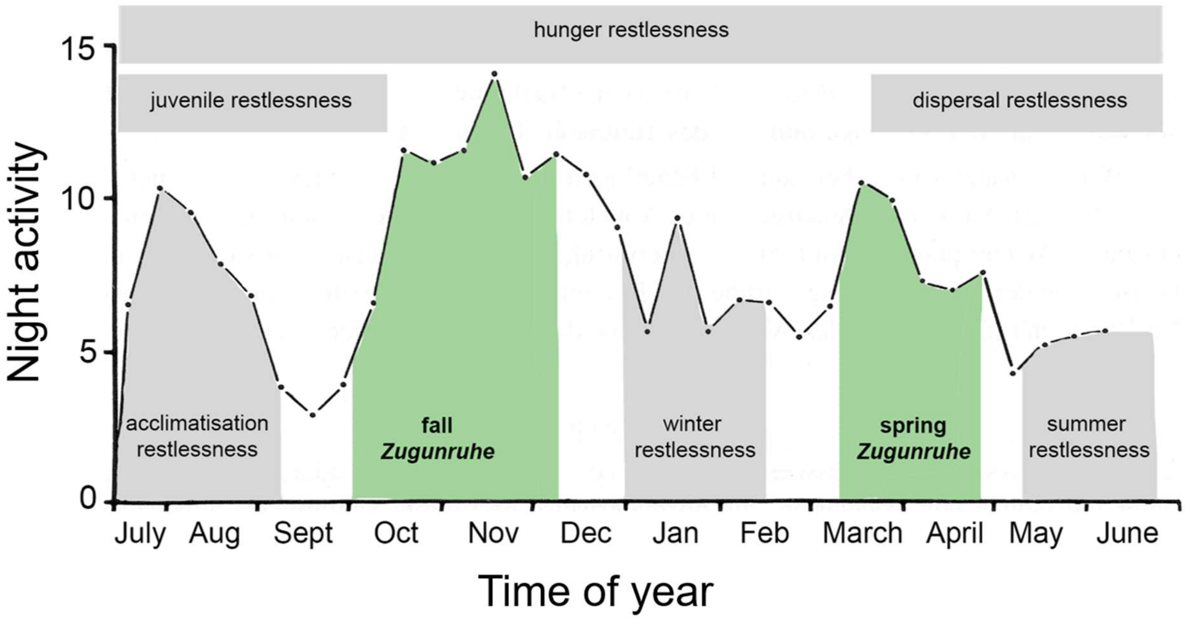

Supplementary Fig. S1: Lability of the avian circadian system. Year-around types of nocturnal restlessness, of which some (bold on green background) relate to seasonal migration. Activity is given has 30 min intervals of night showing activity; after Berthold (1988b).

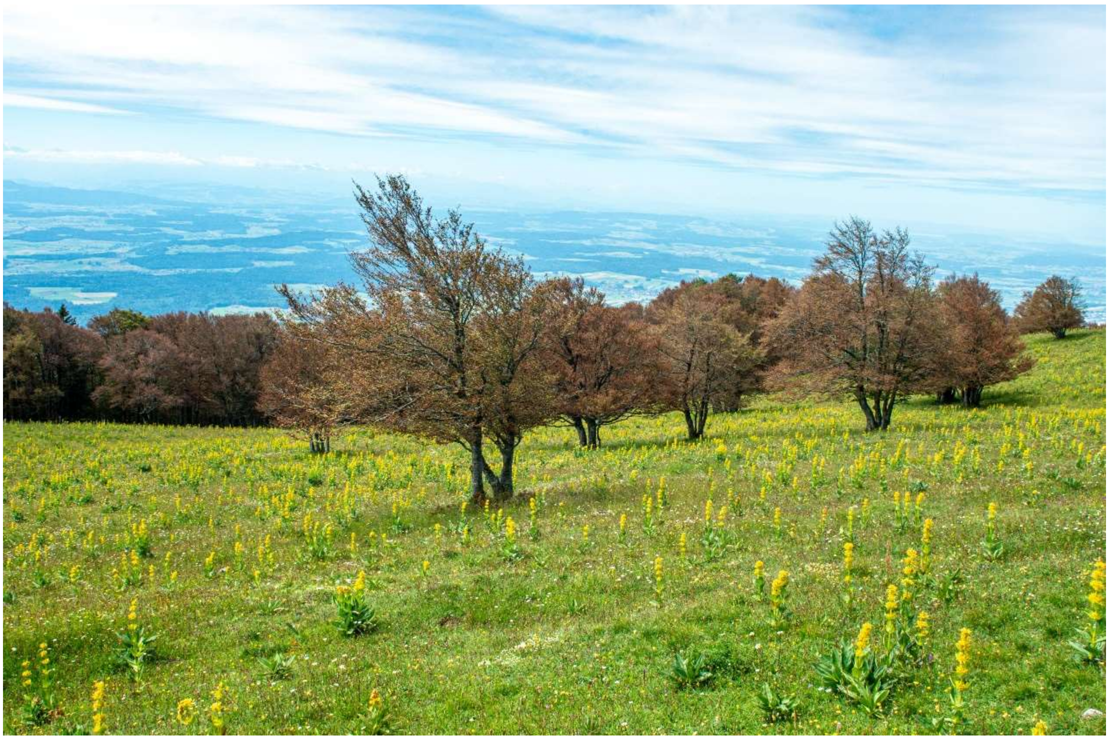

Supplementary Fig. S2: Changing phenology. Due to global warming, spring at higher latitudes steadily advances in most terrestrial habitats. Many organisms also advance phenology, but partly at risk of costs incurred by late winter conditions (top panel showing full canopy defoliation of European beech on 30 June 2020 in the Swiss Jura mountains (1,385m a.s.l; image by Frederik Baumgarten). The frost event occurred on 11 May when leaves started to unfold.

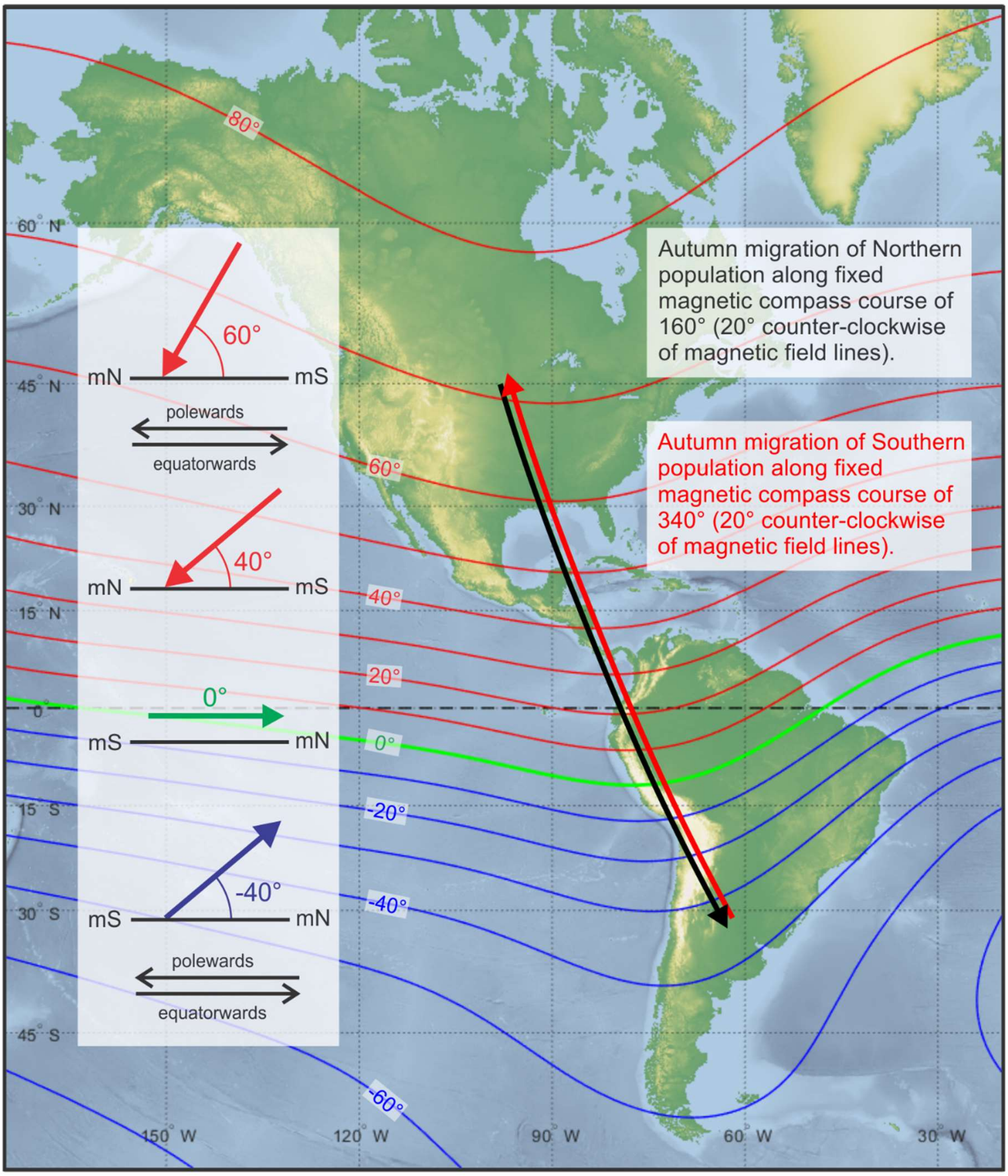

Supplementary Fig. S3: Jointly with re-entrainment, use of a magnetic inclination compass has probably facilitated cross-hemispheric colonization by cliff swallows. The figure shows simulated routes taken by cliff swallows if they follow a constant magnetic compass course. Regardless of whether they start from previous northern or newly established southern breeding grounds, the swallows would reach their respective wintering grounds by the same spatial programme. Tracks are shown against magnetic inclination isolines. The inset on the left shows for selected latitudes the inclination in horitzontal view; abbreviations represent the magnetic directions. Modified after model improvements by Rachel Muheim from Helm and Muheim (2021; see there for details) based on data from (Areta et al. 2021).

Supplementary tables

Table S1. Overview of abbreviations of molecules used in the main text. Indicated are molecules with their full names, followed by aliases in brackets, and a functional note; names follow genecard https://www.genecards.org.

Gene	Full name	Functional note
aa-nat	Aralkylamine N-Acetyltransferase	rhythm-imposing enzyme in melatonin synthesis
adcyap1 (PACAP)	Adenylate Cyclase Activating Polypeptide 1	peptide, glutamatergic regulation of SCN, involved in light resetting; modulates pineal clock genes
bmal1 (arntl1)	Basic Helix-Loop-Helix ARNT Like 1	core clock gene
cAMP	Cyclic adenosine monophosphate	second messenger involved in photic response
ciart (chrono)	Circadian Associated Repressor Of Transcription	clock gene accessory loop
ck1δ,ε (CSNK1D,E)	Casein kinase I isoform delta, epsilon	posttranslational modulator of clock proteins
clock	Circadian Locomoter Output Cycles Protein Kaput	core clock gene
creb1	cAMP Responsive Element Binding Protein 1	transcription factor
dec1,2 (BHLHE4, BHLHE41*)*	differentiated embryo-chondrocyte expressed gene	transcription repressor, e.g., via repressor by binding to E-boxes
cry1,2	Cryptochrome Circadian Regulator 1,2	core clock genes
fos (C-Fos)	Fos Proto-Oncogene, AP-1 Transcription Factor Subunit	immediate (primary) early response gene, involved in communication of photic information to the circadian system
hsf1	Heat Shock Transcription Factor 1	transcription factor mediating thermal response
MAPK1 (ERK2)	Mitogen-Activated Protein Kinase 1	extracellular signal-regulated kinase, regulates upon phosphorylation multiple pathways, e.g., communicating photic cues to circadian system
nfil3 (e4bp4)	Nuclear Factor, Interleukin 3 Regulated	clock gene accessory loop
npas2 (*mop4*)	Neuronal PAS Domain Protein 2	core clock gene
NPY	Neuropeptide Y	neuropeptide involved in food intake regulation and in MAPK communication of photic information to the circadian system
nr1d1,2 (reverbα,β)	Nuclear Receptor Subfamily 1 Group D Member 1,2	nuclear hormone receptor involved in nutrient sensing; clock gene accessory loop
nr3c1 (gr)	Nuclear receptor subfamily 3, group C, member 1	glucocorticoid receptor
nr3c2 (mr)	Nuclear receptor subfamily 3, group C, member 2	mineralocorticoid receptor
OPN1	Iodopsin	photopigment
OPN2	Rhodopsin	photopigment
OPN3	Encephalopsin	photopigment
OPN4	Melanopsin	photopigment
OPN5	Neuropsin	photopigment
per2,3	Period Circadian Regulator 2,3	core clock gene
phlpp1	PH Domain And Leucine Rich Repeat Protein Phosphatase 1	phosphatase involved in light resetting
rorα,β (NR1F1,2)	RAR Related Orphan Receptor A,B	nuclear hormone receptor involved in nutrient sensing; clock gene accessory loop
top1 (topo1)	DNA Topoisomerase I	enzyme altering DNA during transcription, mediating at RORE sites
TRP	transient receptor potential	ion channel superfamily, thermal transducers
VAopsin	Vertebrate ancient opsin	photopigment
